# Facile construction of irinotecan loaded mesoporous nano-formulation with surface-initiated polymerization to improve stimuli-responsive drug delivery for breast cancer therapy

**DOI:** 10.1016/j.heliyon.2023.e15087

**Published:** 2023-04-01

**Authors:** Zheng Nie, Daoliang Wang, Shuangyan Wang, Linling Wang

**Affiliations:** a Department of Breast Surgery, The First People's Hospital of Jingzhou, Jingzhou, 434000, China; b Department of Thyroid and Breast Surgery, The First People's Hospital of Wenling, Wenling, 317500, China; c Department of Ultrasonography, The First Hospital of Hunan University of Chinese Medicine, Changsha, 410000, China

**Keywords:** Irinotecan, Nanotherapy, Mesoporous silica, Breast cancer, Apoptosis

## Abstract

This work uses rice husk to fabricate mesoporous silica nanoparticles (D-RMN) for breast cancer therapy. The biocompatible dual-responsive (DAN-RMN) was developed by polymerizing acrylic acid (AA) and n-isopropyl acrylamide (NIPAM) on the DV-RMN surface monomeric ratio to increase drug delivery efficiency after vinyl groups were added to the surface of nanoparticles (DAN-RMN). Various analytical and spectroscopical methods characterized the fabricated nanoparticles. Additionally, further encapsulation with SN-38 into the DAN-RMN enhances anticancer efficiency. The in-vitro controlled SN-38 release displayed remarkable temperature and pH response. The MTT assay established the biocompatibility and cytotoxicity of natural sources of silica and DAN-RMN. The fabricated SN-38@DAN-RMN nanoparticles effectively killed the MDA-MB-231 and 4T1 cancerous cells, confirmed by the MTT assay. The IC_50_ values of SN-38@DAN-RMN in MDA-MB-231 and 4T1 for 1.8 μg/mL and 1.7 μg/mL, respectively. In addition, acridine orange-ethidium bromide (AO-EB) dual staining methods were used to determine morphological changes of cell shrinkage and fragmentation. Nuclear staining methods confirmed the nuclear fragmentation and condensation of the cells. Further, the cell death was examined using dual staining Annexin V-FITC/PI in flow cytometric analyses to assess apoptosis in the MDA-MB-231 and 4T1 cell lines. The apoptotic cell ratio of SN-38@DAN-RMN in MDA-MB-231 and 4T1 for 27.8 and 32.8, respectively. Since there is no drug leakage in the blood while the carrier is in circulation, the DAN-RMN nanocarrier may be used for targeted and stimuli-responsive administration using ultrasound imaging.

## 1 Introduction

Breast cancer is the second most significant cause of cancer-related mortality in women globally, accounting for more than half of all deaths from the disease [1]. Despite effective early detection and treatment advancements, resistance to traditional chemotherapeutics offers a substantial obstacle to successful breast cancer therapy [[2], [3], [4]]. Metastasis and drug resistance are two critical challenges in breast cancer treatment, which may lead to tumor recurrence and treatment failure [[5], [6], [7]]. As a result, logical therapeutic methods to increase chemotherapy efficacy and improve the clinical outcome of breast cancer patients might be provided by ways to overcome drug resistance [[8], [9], [10]].

The topoisomerase-I inhibitor irinotecan (IRT) was authorized as a first-line treatment for colorectal cancer [11]. A metabolite 7-ethyl-10-hydroxy camptothecin (SN-38) is produced in vivo from IRT, and this metabolite is 2–3 times more cytotoxic than IRT in the presence of cancer cells. Only 2–8% of IRT supplied can be converted to SN-38; substantial dosages of IRT infusions are typically necessary for treatment regimens to achieve therapeutic advantages [[12], [13], [14]]. On the other hand, IRT is linked with severe gastrointestinal toxicity and myelosuppression due to its high dosage. There has been a resurgence of interest in the direct usage of SN-38 due to the unpredictable conversion of IRT to SN-38, the decreased bioavailability of the active ingredient, and the increased risk of adverse effects. Because it is insoluble in water and most pharmaceutically approved solvents and oils, the therapeutic usage of SN-38 remains highly limited [[15], [16], [17]]. Furthermore, SN-38 has a pH-dependent conversion of the lactone ring in acidic settings to the inactive open-form of carboxylate form in neutral or alkaline conditions [18]. Since water-soluble SN-38 is in great demand, efficient drug delivery strategies are required to preserve the active lactone ring of the SN-38 backbone [19].

Targeted drug delivery using nanocarriers has several benefits over traditional small-molecule chemotherapy, including less nonspecific toxicity and increased therapeutic efficiency. Various nanomaterials have been successfully delivered to cancer-targeted medications, liposomes, polymeric nanoparticles, dendrimers, and inorganic-organic hybrid nanoparticles [20]. With their high surface-to-volume ratio, Nanocarriers have the potential to deliver massive doses of anticancer medicines directly to tumor tissues [[21], [22], [23]]. On the other hand, passive and active targeting effects restrict the damaging distribution throughout the system. The aggregation of nanocarriers at tumor locations can be targeted passively using EPR effects to enhance penetration and retention (EPR) [24]. Drug ejection and multiple drug resistance are induced by the absence of cell-specific interactions required to internalize nanocarriers [25]. Nanocarriers can be grafted with antibodies and folic acid to boost further their capacity to target cancer cells, lowering side effects by limiting their interactions with healthy cells, thereby enhancing their ability to target cancer cells [26]. To substantially inhibit tumor growth and minimize skin damage during treatment, we first treat the tumor site with a low-power, concise near-infrared laser [27]. This improves the oxygen concentration while mildly generating a “burning” effect and paves the way for further SDT [28]. Ultrasound (US) can create numerous cavitation bubbles as a high-frequency mechanical vibration wave when acting on live tissue. A powerful shock wave will significantly turbulence local blood in addition to cavitation bubble collapse [29]. As a result, ultrasonography is a useful tool for enhancing blood circulation [30].

The risk of unintended release of the medication during circulation plagues traditional drug carriers. Encapsulating medications before they enter tumors is essential for ideal drug carriers [31]. There are many benefits to using MSNP-based drug delivery systems, including controlling the pore size and surface area while still maintaining the stability of the MSNP-based mesoporous structure. This makes MSNPs an excellent choice for drug delivery [32]. Anticancer medications have been encapsulated in nanopores, and various caps have been employed to seal the pore openings. The cargo in the reservoirs are released into a specified environment when the necessary stimuli are applied [33]. CD-gated MSNPs can effectively store various pore cargoes and release the loaded payloads in response to external stimuli such as redox status, pH changes, enzyme activity, and photoirradiation [34]. Although most of the above systems were examined in solution or in vitro, it is still uncertain how they will perform in vivo [35]. These stimulus-triggered drug release systems have made significant progress. However, it is still difficult to simultaneously achieve high targeting specificity and regulated drug release while preventing nonspecific binding and trapping through the bodies' defense. MSNP-based drug carriers must fulfill the following requirements to be used in vivo. Nanoparticle carriers with high stability in physiologically relevant conditions, 10–100 nm in diameter, and sufficient targeting ligands on their surface to maximize uptake in cancerous cells while reducing nonspecific uptake in off-target cells, and iv) to be capable of removing undesired drug release before achieving tumor, and releasing drugs exceptionally to provide an effect [[36], [37], [38]].

Poly(*N*-isopropyl acrylamide) (PNIPAM), poly(diallyl dimethylammonium chloride) (PDC), poly(allylamine hydrochloride) (PAH), polyetherimide (PEI), and polyacrylic acids (PAA) are only a few of the synthetic polymers often employed in drug administration [39]. While synthetic polymers can accumulate in normal tissues and organs, their potential toxicity must be overlooked. After cellular death or exocytosis, the synthetic polymers cannot be entirely degraded, resulting in their buildup in the tissue [40]. It is possible to hydrolyze or enzymatically break down biodegradable natural polysaccharides into oligosaccharides, di- or monosaccharides, which are then absorbed via biochemical processes [41]. Due to their inherent food properties, dietary polysaccharides are generally considered safe (GRAS) and have excellent biocompatibility and biodegradability. On the other hand, polysaccharides can be used to load and preserve medicines, increasing their therapeutic effectiveness [42].

**Fig. 1:**
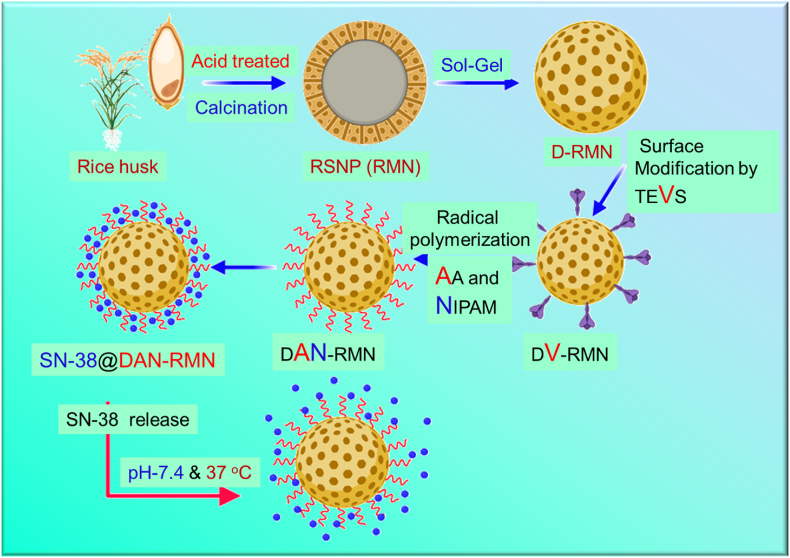
Graphical representation of the DAN-RMN nanocarrier fabrication for treating breast cancer drug delivery.

Biogenic MSNP synthesized from rice husk were accessible to synthesis (RMN), cost-effective, safe, and efficient in various applications [[43], [44], [45]]. The synthetic drug delivery system offers a lower rate of drug release at normal sites, a higher rate at cancerous sites, high drug loading, good dispersion, and excellent biocompatibility due to a well-tuned monomer ratio (Fig. 1). Drug loading and controlled release behavior are being studied novelly in this framework. L929 (non-cancerous) and MDA-MB-231 and 4T1 breast cancer cell lines were used as cancerous and non-cancerous models for a model chemotherapeutic drug irinotecan (SN-38). The L929, MDA-MB-231, and 4T1 cells line examined D-RMN and DAN-RMN for cytotoxicity. Cell morphological and flow cytometric analyses were used to investigate apoptosis as the mechanism of cell death.

## 2 Methods and materials

### 2.1 Materials

The Rice husk used in this research was obtained from the Guangdong Academy of Agricultural Science. Cetyl trimethyl ammonium bromide, sulfuric acid, ammonia solution 25%, hydrochloric acid, Sodium hydroxide, and Dimethyl sulfoxide (DMSO) were obtained from Aladdin (Shanghai, China). Triethoxyvinylsilane, acrylic acid, potassium persulfate, and n-isopropyl acrylamide were obtained from Chemsoon Co., Ltd. (Shanghai, China). Irinotecan (SN-38) was used as received without further purification. MTT (3-[4,5-dimethylthiazol-2-yl]-2,5 diphenyl tetrazolium bromide), fetal bovine serum (FBS), 1% penicillium-streptomycin antibiotic solution, and DMEM medium, and DMEM were purchased from Thermo Fisher Scientific. AO-EB and nuclear DAPI stain were obtained from Sigma-Aldrich (USA). All other chemicals were obtained from Sinopharm Chemical Reagent Co. Ltd. (Shanghai, China).

### 2.2 Characterization

The transmission electron microscopy (TEM) images of fabricated nanoparticles were recorded using a transmission electron microscope (Tecnai F20, FEI company). Field emission scanning electron microscopy (FESEM) and energy-dispersive spectroscopy (EDS) were performed with a Sigma 500 scanning electron microscope (Carl, Germany). The powder X-ray diffraction (XRD) patterns were obtained with X'Pert PRO MPD (Cu-Kα: λ = 1.54 Å). Fourier-transform infrared (FT-IR) spectra were recorded on a PerkinElmer range spectrophotometer with pressed KBr pellets. Dynamic light scattering (DLS) and Zeta plus nano-sizer (Brookhaven Instruments, Santa Barbara, CA, USA) were used to calculate the nanosize and analyze the zeta potential. The nanoparticles' nitrogen adsorption-desorption isotherms were assessed using a TriStar II 3flex (Micromeritics, USA) at 77 K.

### 2.3 Synthesis techniques

#### 2.3.1 Synthesis of D-RMN

To eliminate contaminants, a hydrochloric acid (HCl, 1 M) solution was boiled in rice husks for 4 h. The rice hush (RH) was then rinsed in purified water to remove any remaining acid and dried at 90 °C overnight. To fabricate silica nanoparticles from the rice husk biosource, the dried rice husk was heated to 550 °C for 2 h at 5 °C/min (RSNP). RSNP was dissolved entirely in 7.04 ml of 1 M sodium hydroxide solution to prepare sodium silicate solution (SSS) as the precursor to D-RMN. It was necessary to heat and rapidly stir the prepared mixture to 80 °C until half of the initial volume had evaporated. Preparing D-RMN with a mesoporous structure was first required by dissolving CTAB in 100 ml of deionized water and stirring it at 40 °C for 1 h. For the next 2 h, the SSS was slowly added to a CTAB solution that had been pre-pH- adjusted to 6.5 at 40 °C. Using a 1 M NaOH solution, the pH of the combination was brought up to 11.25, and the mixture was further agitated for an hour. The precipitated material was rinsed with purified water the next day. The last step was drying at 90 °C for 12 h and then calcining at 500 °C for 4 h at 5 °C/min. A new product, D-RMN, was developed [46].

#### 2.3.2 D-RMN surface modification by TEVS to the synthesis of DV-RMN

The vinyl group on the D-RMN was synthesized using the silane coupling agent (TEVS). An ethanol/deionized water solution was treated with ultrasonic waves for 15 min before being added to 40 mL of a 25% ammonia solution and agitated vigorously for 3 h at 27 °C. After that, the solution temperature was increased to 60 °C for an hour. After centrifugation at 8000 rpm for 15 min and three methanol washes, the modified D-RMN was collected. The precipitant that formed was then allowed to dry overnight at a temperature of 40 °C.

#### 2.3.3 Fabrication of DAN-RMN

By using free radical polymerization, DAN-RMN was made After dissolving DV-RMN (100 mg), NIPAM (735 mg), AA (275 μl), and SDS (22.0 mg) in DD-water at 75 °C for 2 h under N_2_ atmospheric condition. The solutions were cooled to room temperature. Once the KPS solution had been made by combining 54 mg of KPS in 5 ml of deionized water, the mixture was stirred for 6 h at 70 °C before the reaction could begin. When dialyzed against deionized water for 3 days, the result yielded DAN-RMN.

### 2.4 Morphological characterization of DAN-RMN

To investigate the DAN-RMN phase transition, 30 mg of nanoparticle was dissolved in 1 ml of pH 5.4 PBS at 4 °C to produce a clear DAN-RMN solution. The temperature of the mixture was then steadily raised. Swelling-to-deswelling transitions were found at a lower critical solution temperature (LCST). Therefore, it was decided to use this temperature to indicate LCST (liquid crystallization temperature). Non-cancerous and cancerous site conditions were considered when conducting the swelling examination. For two days, 0.4 g of the DAN-RMN was submerged in 25 ml of pH 7.2 - PBS containing different solutions at the appropriate temperature and pH. The swelling ratio of the DAN-RMN was measured by using the following equation [47], Swelling ratio = W_t_/W_0_, Whereas Wo and Wt are the weights of dry and swollen NPs at zero and time t, respectively.

### 2.5 Drug loading into the nanoparticles

The incorporation process demands the drug to be incorporated at the time of nanoparticle formulation. Deionized water dissolved 35 mg of D-RMN or DAN-RMN in 3 ml of deionized water at 4 °C. In the following 24 h, the mixture was incubated at 4 °C with 2.5 ml of Irinotecan (3 mg/ml). The solution was centrifuged at 8000 rpm for 3 min and twice rinsed with DD-water to attain the SN-38@D-RMN and DAN-RMN. The amount of drug loaded into the two D-RMNs using a UV spectrophotometer was detected at a wavelength of 482 nm [48].

### 2.6 *In vitro* drug release behavior

Nanoparticles' in vitro release kinetics offer important evidence concerning their capacity to modify drug release. Thus, it is crucial to study the safety, efficacy, and quality of the drugs/nanoparticles. D-RMN and DAN-RMN were distributed in 6 ml of PBS with different pH values (7.4 and 5.4) to examine whether nanocarriers release drugs. When the dispersions reached the required temperature, they were put into a shaker incubator. Afterward, the mixtures were centrifuged, and the supernatant solution was replenished with new PBS at predetermined intervals [48]. The drug-release amount was calculated using a UV spectrophotometer detected at 482 nm.

### 2.7 Cell culture

Human breast cancer cells (MDA-MB-231 and 4T1) and Human fibroblast cells (L929, non-cancerous cell) were obtained from the Cell Bank of the Chinese Academy of Sciences (Shanghai, China). Cancer and non-cancerous cells were maintained in Dulbecco's modified Eagle's medium (DMEM) supplemented with 10% fetal bovine serum (FBS) in a humidified atmosphere at 37 °C. The cell population (80−90%) was harvested using trypsin, washed in PBS, and used for further experiments.

### 2.8 Cytotoxicity examination

Cell cytotoxicity suggests the capability of certain drugs/nanoparticles or mediator cells to damage living cells. The MDA-MB-231 and 4T1 cancerous cells and L929 non-cancerous fibroblast cells were purchased from KeyGEN BioTECH Co., Ltd. and cultured in Dulbecco's modified Eagle's medium (DMEM) containing 10% of fetal bovine serum (FBS). The cytotoxicity of D-RMN, DAN-RMN, SN-38@D-RMN, and SN-38@DAN-RMN was evaluated on non-cancerous L929 cells and cancerous MDA-MB-231 and 4T1 by standard methyl thiazolyl tetrazolium (MTT) assay. The viability of the L929 cells was examined, and the cytotoxicity of the MDA-MB-231 and 4T1 cells was treated with SN-38@D-RMN and SN-38@DAN-RMN (0.4–200 μg/mL) nanoparticles. Briefly, cells were seeded into 96-well plates at the cell density of 1 × 10^3^ cells per well in 200 μL per well medium and cultured for 24 h. Then, the medium in all wells was replaced with fresh ones containing different concentrations of the nanoparticles (0.08–20 μg/mL), and the cells were cultured for another 24 h. After that, MTT (5 mg/mL) was added to the culture medium in each well and incubated for an additional 4 h. Finally, each well was washed with sterile PBS thrice, and the medium was replaced with 150 μL DMSO to dissolve the residues. Thermo MK3 ELISA reader measured each well's optical density at 570 nm to access cell viability [[49], [50], [51], [52], [53]]. Three independent experiments were conducted for each concentration, and four replicates were performed in each independent investigation.

### 2.9 Morphological evaluation of the apoptotic cells

Biochemical staining assays confirmed the morphological damage of the cells. Free SN-38, SN-38@D-RMN, and SN-38@DAN-RMN -treated MDA-MB-231 and 4T1 cells were investigated by AO/EB fluorescence staining techniques to determine apoptosis. MDA-MB-231 and 4T1 cells were cultured for 24 h in 6-well plates at a density (1 × 10^3^) of cells per well in 200 μL per well medium. The cells were then treated for 24 h to the determined inhibitory concentration (IC_25_). The control cells were left untreated. Combining the two dyes designed at 100 μg/mL in PBS each, a combination of the AO/EB dyes (20 μL) was added [[54], [55], [56], [57]]. These cells were tracked and observed using a fluorescence microscope after staining the treated and control samples (Olympus CKX-53).

Free SN-38, SN-38@D-RMN, and SN-38@DAN-RMN -treated MDA-MB-231 and 4T1 cells were investigated by nuclear fluorescence stain DAPI techniques to determine apoptosis. MDA-MB-231 and 4T1 cells were cultured for 24 h in 6-well plates at a density (1 × 10^3^) of cells per well in 200 μL per well medium. The cells were then treated for 24 h to the determined inhibitory concentration (IC_25_). The control cells were left untreated. Combining the two dyes designed at 100 μg/mL in PBS each, a combination of the DAPI nuclear dye (20 μL) was added [58]. These cells were tracked and observed using a fluorescence microscope after staining the treated and control samples (Olympus CKX-53).

### 2.10 Cell apoptosis investigation

The Annexin V-FITC/PI double staining test was used to assess the degree of apoptosis. MDA-MB-231 and 4T1 cells were cultured for 24 h in 6-well plates at a density (3 × 10^3^) of cells per well in 200 μL per well medium. The treatment was limited to the IC_25_ concentrations of free SN-38, SN-38@D-RMN, and SN-38@DAN-RMN. Briefly, the obtained cells were washed in PBS at least twice before stained with PI and Annexin V-FITC. Flow cytometry was used (Millipore Corporation, Billerica, MA, USA). Direct counting of the cells identified the number of living, necrotic, late apoptotic, and early apoptotic cells [56].

## 3 Results and discussion

### 3.1 Fabrication methods and characterization

**Fig. 2:**
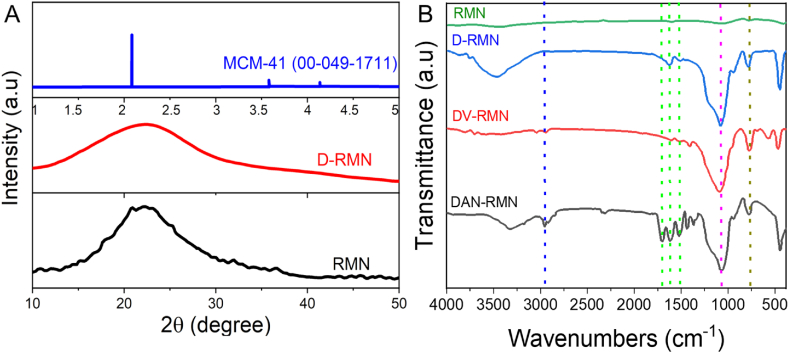
Characterization of silica nanoparticles. A) X-ray diffraction (XRD) patterns of RMN, D-RMN and compared with MCM-41 (00-049-1711). B) FT-IR spectral analysis of RMN, D-RMN, D-vinyl-RMN, and DAN-RMN.

Fig. 1 depicts the whole technique for synthesizing the DAN-RMN. Bio-silica nanoparticles derived from rice husk were used to precursor the sol-gel method to produce MSNs from biogenic sources. Wide-angle XRD patterns from the RSNP are shown in Fig. 2A. It was easy to spot silica's amorphous form by its distinctive broadband at 22.17° and 2ɵ angle. َ Furthermore, the profile revealed no contaminants. D-low RMN's angle XRD pattern is shown in Fig. 2A. As expected, the nanomaterials with high crystallinity had a strong peak in the XRD pattern at 2 = 2.08°, which indicates the particles' hexagonal phase.

**Fig. 3:**
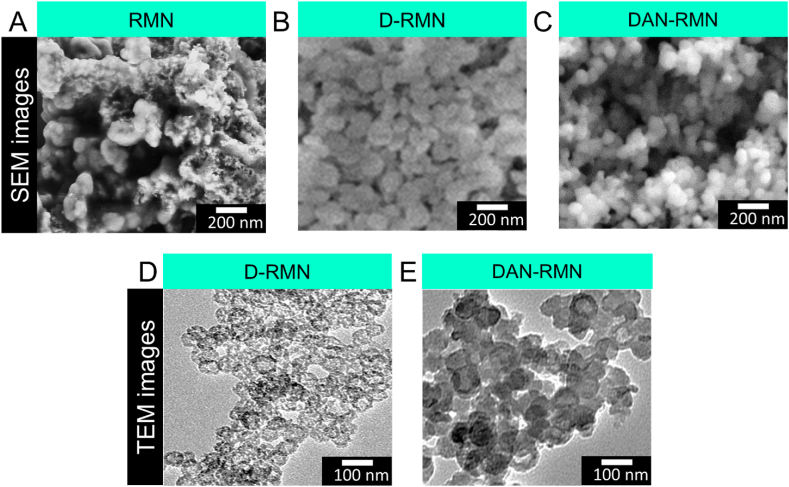
A–E) Morphological characterization of nanoparticles. Scanning electron miscopy (SEM) images of RMN, D-RMN, and DAN-RMN. Transmission electron miscopy (TEM) images of D-RMN and DAN-RMN.

**Fig. 4:**
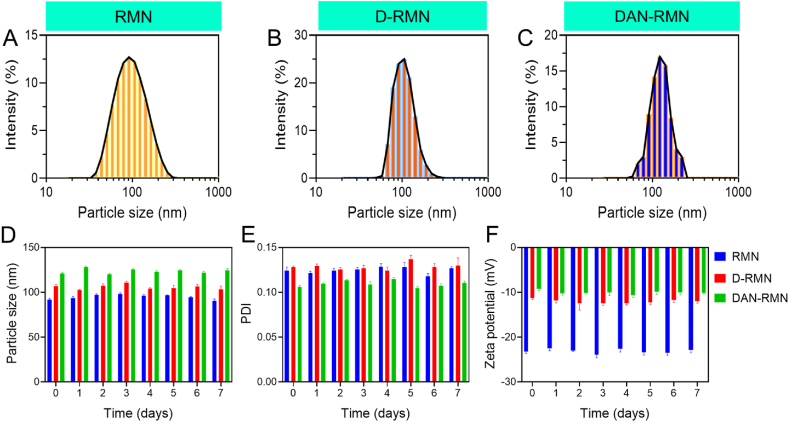
A–C) The hydrodynamic parameters of different nanomaterials. DLS measured the size distribution of RMN, D-RMN, and DAN-RMN. Aqueous solutions at room temperature examined the stability of the RMN, D-RMN, and DAN-RMN. D) particle size. E) Polydispertive index (PDI). F) Zeta potential. All the investigations were measured by DLS measurement. Data are presented as mean ± standard deviation (SD).

Nanoparticles were analyzed using FT-IR to ensure that the production of the nanoparticles was proper. The symmetric and asymmetric stretching vibration of -*Si*-*O*-Si- was shown in Fig. 2B as distinct bands at 462 cm^−1^, 812 cm^−1^, and 1103 cm^−1^. The 3452 cm^−1^ peaks were caused by symmetric frequency stretching of the -SiOH band. The surface intensity of the silanol group dropped dramatically during the D-RMN surface vinylation process. At 1623 cm^−1^, the OH stretching vibration of H_2_O overlapped the CC group of VTES. The CH of vinyl groups was also related to the 2977 cm^−1^ band. We need to observe distinct bands of polymers on the D-RMN surface to establish whether the polymers have bound to the surface. NIPAM N-H and C-N stretching bands were visible at 1542 and 1456 cm^−1^, respectively, whereas the amide group in NIPAM emitted a 1386 cm^−1^ signal [59]. The CO signal peaked at 1726 cm^−1^ and 1543 cm^−1^, while the CC signal peaked at 1640 cm^−1^. There were peaks at 2974 cm^−1^ and 1255 cm^−1^ due to the NIPAM isopropyl group's C-H band [59]. Scanning electron microscopy (SEM) and transmission electron microscopy (TEM) were used to examine the nanoparticles' morphology and size. There was a spherical form to the RSNP, the D-RMN, and the DAN-RMN (Fig. 3A–E). The mean size of RSNP, D-RMN, and DAN-RMN was 91.3, 102.1, and 122.3 nm (Fig. 4A–C), and the PDI of RSNP, D-RMN, and DAN-RMN was 0.121 and 0.127, and 0.105 respectively, showing a uniform distribution. The zeta potential of RSNP, D-RMN, and DAN-RMN was around −23.1, −11.6, and −9.4 mV. The stability of the RSNP, D-RMN, and DAN-RMN was examined for the dynamic light scattering (DLS) measurement for 7 days with an aqueous solution at room temperature. The particle size (Fig. 4D), PDI (Fig. 4E), and zeta potential (Fig. 4F) show that slightly significant changes were found after seven days of the experiment.

**Fig. 5:**
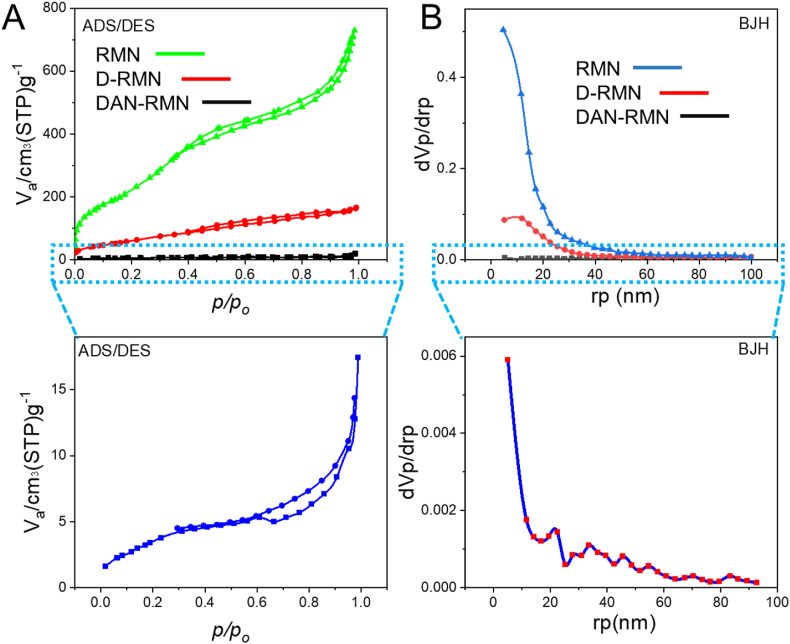
The nitrogen adsorption-desorption isotherms (**A**) and BJH pore size distributions (**B**) of samples RMN, D-RMN, and DAN-RMN.

With CTAB as a template, the RMN was developed with a minimal surface region and suitable overall volume pore. Fig. 5A and B demonstrated that the pore size distributions and the N2 adsorption/desorption isotherms of RSNP, D-RMN, and DAN-RMN were all shown. The IUPAC categorization of isotherms has validated a mesoporous structure as type IV. The surface areas of RSNP and D-RMN were 227.43 cm^2^ g^−1^ and 951.25 cm^2^ g^−1^. Also, they had an average volume pore of 0.2499 cm^3^ g^−1^ and 1.1219 cm^3^ g^−1^, respectively. The RSNP and D-RMN exhibited a small pore size to the related pore size distribution curve (Fig. 5). RSNP and D-RMN had pore diameters of 4.41 and 4.72 nm, respectively. As a result, it is impossible to quantify DAN-surface RMN 's area, pore volume, or pore diameter since the D-RMN is entirely imprisoned in the polymeric shell. As shown in Fig. 5, the DAN-RMN N2 adsorption-desorption values are undetectable. Phase transition of DAN-RMN was used to identify the LCST point. The solution turns opaque from clear at a temperature of 38 °C. In both the body (pH = 7.3 and 37 °C) and cancerous location (pH = 5.4 and 40 °C), DAN-RMN edema was examined (Fig. 5). The swelling ratio for 48 h was 225% and 37%, respectively. These nanoparticles, therefore, have a higher rate of swelling in normal tissues, which acts as an intelligent gatekeeper and prevents medications from being released prematurely. Although DAN-RMN was in a collapsed state, the polymeric shell allowed chemotherapeutics to enter the cancerous location.

### 3.2 *In-vitro* SN-38 release

**Fig. 6:**
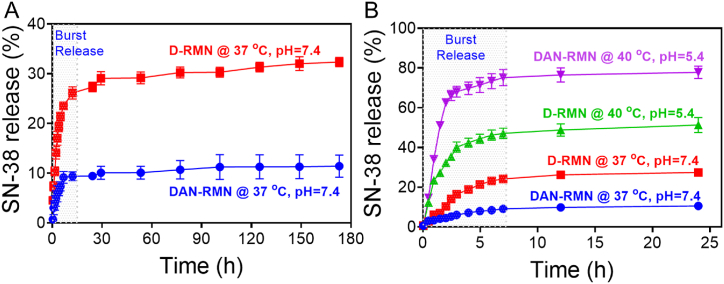
In vitro SN-38 release profile. A) Long-term examination of different pH (pH = 5.4 and 7.4) and different temperatures (37 °C and 40 °C). B) SN-38 release profiles from SN-38@D-RMN and SN-38@DAN-RMN at 37 °C for pH = 7.4 for 24 h. Data are presented as mean ± standard deviation (SD).

Shaking SN-38-loaded D-RMN and DAN-RMN at 37 °C and 40 °C in the PBS with various pH values of 7.4 and 5.4 to examine the in-vitro SN-38 release behavior is shown in Fig. 6A. At a pH of 5.4, nanoparticles released drugs more quickly than at a pH of 7.4, which, on the solubility of SN-38 in D-RMN, increases the SN-38 release rate. Protonation of the SN-38 amine group increases when the pH is lower. Thereby, SN-38's hydrophilicity was improved so that the D-RMN could be released quickly. As shown in Fig. 6B, polymers have enhanced the drug release ratio in two pH values of 7.4 and 5.4. At pH 7.4, the –COOH groups attracted the positive charge on SN-38 with a significant electrostatic force. This is why the release of SN 38 was limited by the DAN-RMN, as stated above. The protonation of the carboxylic acid group was achieved by lowering the pH to 5.4, which reduced the electrostatic contact and resulted in a fast release of the medication. The LCST of NIPAM is generally accepted to be 32 °C. A cancerous location necessitates that the LCST be perfected for a controlled drug release profile. The copolymerization of NIPAM can alter the LCST with other monomers, such as AA. The copolymeric LCST is improved by raising the AA content during polymerization. It is vital to enhance the LCST over 37 °C since cancerous sites are higher in temperature than normal tissues. The LCST used in this study was calibrated to 38 °C. This is because the nanoparticle shell of the PNIPAM was in a collapsed hydrophobic condition when the temperature above the DAN-RMN LCST limit of 40 °C permitted the aqueous-soluble SN-38 to disperse out of NPs shell rapidly. The controlled drug release from DAN-RMN was around 9.1% in 24 h and 10.2% for 7 days, much less than that of D-RMN (26.6% in 24 h and 31.7% over 7 days) at pH 7.4 and 37 °C, which matched the body state. Drug release efficiency in cancerous locations was enhanced, while the total amount of pharmaceuticals released in healthy tissues decreased due to the DAN-RMN.

### 3.3 MTT assay

**Fig. 7:**
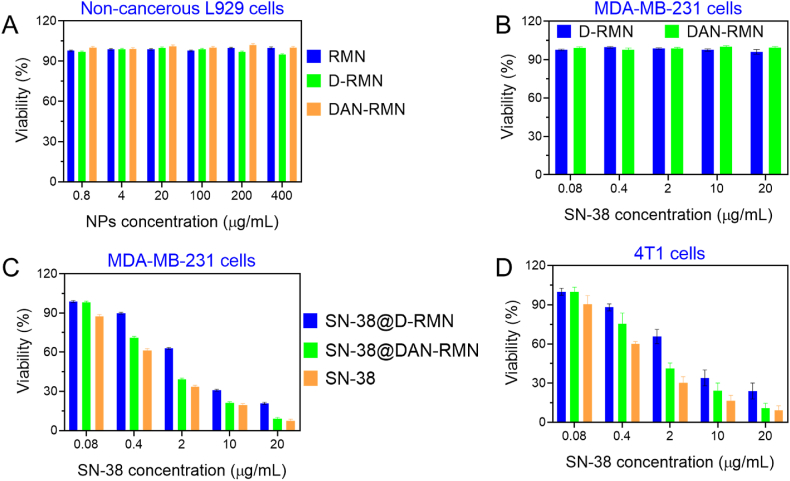
In vitro cytotoxicity examination. The SN-38, SN-38@D-RMN, and SN-38@DAN-RMN were treated with L929 non-cancerous cells, MDA-MB-231, and 4T1 breast cancer cells. A) L929 cells treated with RMN, D-RMN, and DAN-RMN. B) MDA-MB-231 cells treated with D-RMN and DAN-RMN. C-D) MDA-MB-231 and 4T1 cells treated with SN-38, SN-38@D-RMN, and SN-38@DAN-RMN. Data are presented as mean ± standard deviation (SD).

The MTT analysis examined the viability of L929 fibroblast cells with nanoparticle doses ranging from 0.8 to 400 μg/mL to analyze blank nanoparticles' in-vitro cytotoxicity and biocompatibility. Fig. 7A shows the toxicity of RSNP, D-RMN, and DAN-RMN. RSNP was shown to be a safe and biocompatible source of silica. No substantial toxicity of the nanoparticles was found due to the non-toxic profile of silica and biocompatibility polymers. D-RMN and DAN-RMN were shown to have excellent biocompatibility based on their synthesis from biosource and their modification with biocompatible polymers. Fig. 7B–D was used to evaluate the potential of SN-38@D-RMN and SN-38@DAN-RMN as drug delivery systems for cancer treatment. Because of its greater release efficiency, SN-38@DAN-RMN was more hazardous than SN-38@D-RMN at the same SN-38 doses. The SN-38@DAN-RMN impact on MDA-MB-231 and 4T1 was comparable to the SN-38 toxicity effect on the cell line. This device can serve as a high-efficiency means of delivering drugs for cancer treatment. The blank D-RMN and DAN-RMN were safe for the MDA-MB-231 and 4T1 cell lines. Yan Li et al. developed mesoporous nanoparticles encapsulated with folic acid to enhance breast cancer cell lines, revealing less anticancer activity compared to our results [38].

### 3.4 Morphological assay and mode of cell death

**Fig. 8:**
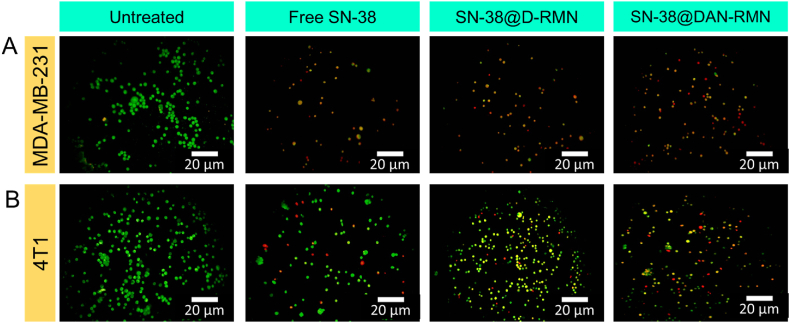
A-B) Fluorescence microscopic images of AO-EB dual staining. MDA-MB-231 and 4T1 cells were treated with IC_25_ concentrations of SN-38, SN-38@D-RMN, and SN-38@DAN-RMN. Scale bar 20 μm. The green color represents the live cells, and the red and reddish-orange color reveals the apoptotic cells. (For interpretation of the references to color in this figure legend, the reader is referred to the Web version of this article.)

**Fig. 9:**
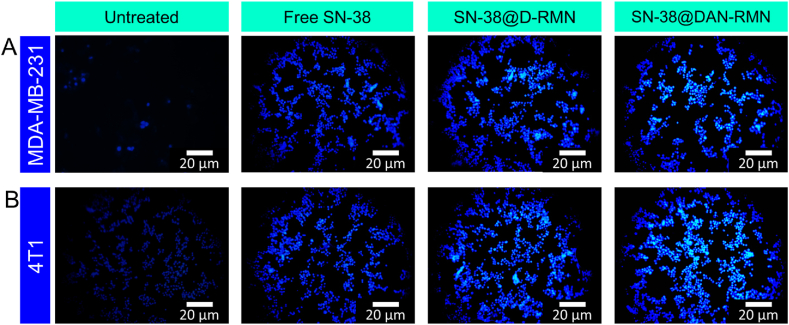
A-B) Fluorescence microscopic images of DAPI nuclear staining. MDA-MB-231 and 4T1 cells were treated with IC_25_ concentrations of SN-38, SN-38@D-RMN, and SN-38@DAN-RMN. Scale bar 20 μm. The blue color represents the live cells, and the dark blue color reveals DNA fragmentation and condensation. (For interpretation of the references to color in this figure legend, the reader is referred to the Web version of this article.)

**Fig. 10:**
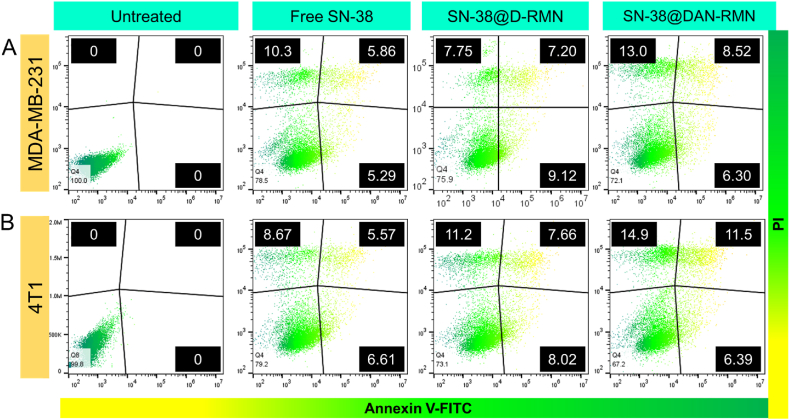
A-B) Apoptosis induction was examined by Annexin-V FITC/PI staining. Flow cytometry data showed a superior level of apoptosis ratio was detected in MDA-MB-231 and 4T1 breast cells. MDA-MB-231 and 4T1 cells were treated with IC_25_ concentrations of SN-38, SN-38@D-RMN, and SN-38@DAN-RMN.

According to several studies, nanoparticle inhibition of cancer cells is associated with apoptosis. As a result, we believe nanoparticles can inhibit cancer by increasing cell death [60]. Apoptosis is a genetically regulated programmed cell death that keeps organisms in balance. In healthy cells, apoptosis is a natural and orderly process. Cancer cells, on the other hand, may survive apoptosis and divide. Anticancer drugs work by causing apoptosis, which kills cancer cells. Therefore, the apoptosis of breast cancer MDA-MB-231 and 4T1 cells treated with SN-38@D-RMN and SN-38@DAN-RMN nanoparticles for 24 h was observed by morphological changes in staining and flow cytometry, as shown in Fig. 8, Fig. 9, Fig. 10. This tendency was consistent with the cytotoxicity data, showing that cell apoptosis is SN-38@D-RMN and SN-38@DAN-RMN at IC_25_ concentration.

The AO/EB fluorescence microscopic staining assay observed the morphological differences in MDA-MB-231 and 4T1 cells. AO/EB staining distinguishes between live and apoptotic/necrotic cells. Fig. 8A and B illustrates the untreated (control), SN-38@D-RMN, and SN-38@DAN-RMN -treated cells at IC_25_ concentrations after 24 h. The color of the control cells remained green after staining, but the color changed after the treated cells (orange), suggesting the presence of apoptotic cells. The treated cells also have nuclear disintegration, shrinkage, and membrane blebbing. The results obtained were in good agreement with the cytotoxicity findings. Nihal S. Elbialy's co-workers developed the mesoporous nanoparticles with curcumin and investigated the cell apoptosis for HepG2 at time points 24 h and 48 h post incubation was mild. The above results agree with previous studies [36].

The outcome of SN-38@D-RMN and SN-38@DAN-RMN on nuclear changes was observed using the DAPI staining assay. Compared to the untreated control, DAPI staining of SN-38@D-RMN and SN-38@DAN-RMN-treated cells for 24 h revealed substantial alterations in the shape of the chromatin nuclear (Fig. 9A and B). The treated cells exhibit a dazzling blue hue, abnormal nuclei, condensed chromatin, and an irregular cell shape, whereas control cells have typical spherical nuclei and a conventional blue color. Our findings are consistent with prior research on the effect of nanoparticles on apoptosis in MDA-MB-231 and 4T1 cells. The apoptosis was further confirmed with Annexin V/PI flow cytometric assay.

Apoptosis, autophagy, and necrosis are the significant types of apoptosis [61]. To further confirm cell death, an Annexin V/PI staining assay was used. The assay revealed the apoptosis in MDA-MB-231 and 4T1 cancer cells exposed to IC_25_ concentrations of SN-38@D-RMN and SN-38@DAN-RMN for 24 h. Untreated cells showed no substantial apoptosis, as shown in Fig. 10A and B. SN-38@D-RMN and SN-38@DAN-RMN-treated cells, on the other hand, become apoptotic after 24 h, with apoptotic cell populations of 29.5% and apoptotic cell populations of 33.5%, respectively. Changes in the population of viable cells show that the cell becomes apoptotic due to the anticancer actions of SN-38@D-RMN and SN-38@DAN-RMN. The composite nanoparticles prepared in this work display a comparable cytotoxic effect against breast cancer cells to previously reported MSNs loaded with SN-38.

On MDA-MB-231 and 4T1 cancer cells, an AO/EB dual staining assay was carried out with newly fabricated nanoparticles. Before being stained, the cells were treated for 24-h with SN-38, SN-38@D-RMN, and SN-38@DAN-RMN. Numerous greenish-yellow cells with granulated nuclei were found in SN-38-loaded targeted MSNPs, indicating that these cells were going through apoptosis [62]. Dead cells exhibited red fluorescence because of EB intercalation in their DNA [55]. As a result of the decreased cellular absorption of bare MSNPs, most of the cells treated with SN-38-loaded MSNPs were green and had normal nuclei morphology, indicating that they were alive. This work has made it easier to identify apoptosis in cells that have been exposed to targeted NPs that are loaded with SN-38. It has also accurately distinguished various phases of apoptosis and caused morphological alterations. These data indicate that the DOX-loaded mesoporous nanoparticles caused apoptosis in the MCF-7 cells [59].

Cells labeled with PI and Annexin V-FITC showed early and late apoptosis [[63], [64], [65]]. After the start of apoptosis, phosphatidylserine, found in cells' inner membranes, is translocated to the surface of the plasma membrane. Damaged and dead cells are marked by PI, whereas Annexin V coupled to a fluorescent dye readily binds to the exposed phosphatidylserine. Taken together, we revealed that SN-38@D-RMN and SN-38@DAN-RMN based on morphological changes apoptosis induction could increase mortality in breast cancer cells.

Taken together from the overall in vitro analysis (MTT assay, staining methods, and flow cytometry analysis), next we move on to the in vivo systemic toxicity in the animal model, antitumor efficacy, pharmacokinetics, and specific target imaging techniques with NIR fluorescence technique. In this work, nanoparticle stability, biocompatibility, and dehydration are not limited. The newly fabricated nanoparticles are highly suitable for the animal model without side effects.

## 4 Conclusion

The present work synthesized an affordable, efficient, biocompatible, and dual-responsive drug delivery system. Biologically active mesoporous silica nanoparticles were produced from rice husk, then AA and NIPAM monomeric units were grafted on a modified DV-RMN surface. The results reveal the temperature/pH-responsive copolymer shell of the DAN-RMN can govern the opening and shutting of the pores. The cytotoxic activity and inducing apoptosis of the DAN-RMN were examined on the L929 non-cancerous and MDA-MB-231 and 4T1 cells as model normal and malignant cells. The biocompatibility and viability of the source and DAN-RMN were established using the MTT test on the L929 cell. SN-38@DAN-RMN showed cytotoxic activity like free SN-38 to MDA-MB-231 and 4T1 cell lines. The different biochemical staining methods confirmed the morphological variations and nuclear DAPI staining methods, and the flow cytometric analysis revealed the mode of cell death in MDA-MB-231 and 4T1 breast cancer cells. Overall, the as-prepared SN-38@DAN-RMN demonstrated delightful biocompatibility and dual temperature/pH responsiveness, delivering a new paradigm for effective clinical tumor treatment. In the future, these multifunctional nanocomposites have been shown to effectively restrain tumor growth based on improved and synergistic chemotherapy and ultrasound ablation, offering useful nanoplatforms for breast cancer treatment.

## Author contribution statement

Zheng Nie: Performed the experiments; Analyzed and interpreted the data; Wrote the paper.

Daoliang Wang: Conceived and designed the experiments; Contributed reagents, materials, analysis tools or data.

Shuangyan Wang, Linling Wang: Conceived and designed the experiments.

## Funding statement

This research did not receive any specific grant from funding agencies in the public, commercial, or not-for-profit sectors.

## Data availability statement

Data will be made available on request.

## Declaration of competing interest

The authors declare that they have no known competing financial interests or personal relationships that could have appeared to influence the work reported in this paper.
